# Using historical data to facilitate clinical prevention trials in Alzheimer disease? An analysis of longitudinal MCI (mild cognitive impairment) data sets

**DOI:** 10.1186/s13195-021-00832-5

**Published:** 2021-05-07

**Authors:** Manfred Berres, Andreas U. Monsch, René Spiegel

**Affiliations:** 1 University of Applied Sciences Koblenz, Koblenz, Germany; 2 University Department of Geriatric Medicine FELIX PLATTER, Basel, Switzerland

**Keywords:** Historical controls, MCI criteria, Clinical trial, Cohort study, Convenience sample, Meta-analysis

## Abstract

**Background:**

The Placebo Group Simulation Approach (PGSA) aims at partially replacing randomized placebo-controlled trials (RPCTs), making use of data from historical control groups in order to decrease the needed number of study participants exposed to lengthy placebo treatment. PGSA algorithms to create virtual control groups were originally derived from mild cognitive impairment (MCI) data of the Alzheimer’s Disease Neuroimaging Initiative (ADNI) database. To produce more generalizable algorithms, we aimed to compile five different MCI databases in a heuristic manner to create a “standard control algorithm” for use in future clinical trials.

**Methods:**

We compared data from two North American cohort studies (*n*=395 and 4328, respectively), one company-sponsored international clinical drug trial (*n*=831) and two convenience patient samples, one from Germany (*n*=726), and one from Switzerland (*n*=1558).

**Results:**

Despite differences between the five MCI samples regarding inclusion and exclusion criteria, their baseline demographic and cognitive performance data varied less than expected. However, the five samples differed markedly with regard to their subsequent cognitive performance and clinical development: (1) MCI patients from the drug trial did not deteriorate on verbal fluency over 3 years, whereas patients in the other samples did; (2) relatively few patients from the drug trial progressed from MCI to dementia (about 10% after 4 years), in contrast to the other four samples with progression rates over 30%.

**Conclusion:**

Conventional MCI criteria were insufficient to allow for the creation of well-defined and internationally comparable samples of MCI patients. More recently published criteria for MCI or “MCI due to AD” are unlikely to remedy this situation. The Alzheimer scientific community needs to agree on a standard set of neuropsychological tests including appropriate selection criteria to make MCI a scientifically more useful concept. Patient data from different sources would then be comparable, and the scientific merits of algorithm-based study designs such as the PGSA could be properly assessed.

## Introduction

Almost 10 years ago, our group published the PGSA (Placebo Group Simulation Approach) for debate to the Alzheimer community [1]. The proposed novel study design was intended to partially substitute for RPCTs (randomized placebo-controlled trials), i.e., clinical studies which by definition expose some of the participants to treatment with placebo. We argued that, in the case of Alzheimer’s disease (AD), clinical prevention trials with pre-clinical subjects would typically last 18 months or longer—and that it was ethically problematic to put individuals with a high risk of developing dementia on an a priori inactive long-term medication. Instead of a concomitant placebo group, the PGSA introduced algorithm-based forecasts of trials subjects’ expected own disease trajectories to account for the effects of baseline differences, time in the study, and the circumstances of trial participation. The original PGSA algorithms were derived from the Alzheimer’s Disease Neuroimaging Initiative (ADNI) data [2] available at that time, i.e., from a then recent but, nonetheless, historical data set.

The pros and cons of using historical data in clinical trials have been discussed under a number of aspects [3]. Thus, in the case of rare diseases, it may be difficult to recruit sufficient numbers of patients for proper control groups in addition to the treatment group [4]. In the case of progressive, non-reversible and potentially fatal diseases, there are ethical issues limiting the use of inactive treatment, and it may also be difficult to obtain consent from patients for participation if one of the treatment options looks more promising than the other one. Finally, in situations where no effective treatments are available and a promising candidate treatment is to be tested, it may be difficult to convince all potential trial subjects to participate in a study which includes a placebo arm [5]. However, the absence of certain subgroups of patients in a clinical trial will lead to less representative samples and is then likely to cause bias in the results. Historical data could be considered in some of these situations as a potential substitute for a concomitant control group (for ALS see [6]).

A necessary prerequisite for using historical controls is that they are comparable to the current study population. This requirement is hardly ever fulfilled. If only demographic variables such as age, sex, and observable health status are different, adjustments for these covariates might solve the problem. If one attempts to set up a model for such adjustments, several potential historical controls need to be compared [7].

In the specific case of mild cognitive impairment (MCI), a clinical condition between normal aging and dementia (see definition in [8]), the problem of finding adequate historical data is aggravated by the fact that MCI criteria have shifted over time and that there is no rigorous and generally accepted definition of the condition [9–11]. As a typical consequence thereof, different clinical drug trials with MCI subjects in the past have applied different inclusion and exclusion criteria [12]. In this paper, we investigate whether information from large MCI databases can be summarized in a heuristic manner such that a “standard control group” for use in future clinical trials with this population could be created. We will compare data from two cohort studies, one clinical drug trial and two convenience patient samples to investigate:

- Inclusion and exclusion criteria for MCI applied in five different patient datasets
- The selection of cognitive tests applied at study entry and at follow-up
- The homogeneity of patients’ demographic and baseline data
- The homogeneity of disease progression as measured by cognitive tests and indicated by the proportion of transitions from MCI to dementia

While distributions of demographic and baseline data will be shown, the progression of the disorder is analyzed as the “effect of no treatment over time”. In a clinical study, this corresponds to the progression observed in the control group and would be contrasted to the progression observed in the treatment group. In the five datasets considered, an overall judgement was provided at each patient visit by an experienced clinician as to whether the patient had progressed from MCI to dementia. Progression rates and hazard ratios will be compared between studies.

In all the studies considered in this analysis, and in particular in the clinical drug trial [13], some patients were treated with anti-Alzheimer medication such as cholinesterase inhibitors or memantine. While these drugs are considered transiently effective in AD [14], none of them was shown to have significant and maintained effects on the progression of the disease from MCI to dementia [8, 13, 15]. For this reason, we will consider all subjects as “untreated” in our analyses.

## Material and methods

### Datasets used

We analyzed individual patient data from the following:

- The Alzheimer‘s Disease Neuroimaging Initiative (ADNI; http://www.loni.ucla.edu/ADNI)
- The National Alzheimer’s Coordinating Centers (NACC; https://www.alz.washington.edu)
- The InDDEx clinical trial [13]
- The German Dementia Competence Network (CNG [16];)
- The Basel University Memory Clinic (BS-MC)

The ADNI and the NACC samples included only patients who were between 54 and 90 years old at entry. BS-MC included only patients with at least 7 years of education. In order to reduce variability, these restrictions were applied to all 5 datasets in our analyses.

The ADNI (Alzheimer’s Disease Neuroimaging Initiative) study aims at investigating the prognostic value of biomarkers, in particular of MRI and PET images, to describe the progression of Alzheimer’s disease from its preclinical to its symptomatic stages. It is led by the principal investigator [2] and representatives of the ADNI sites, the NIH (National Institutes of Health), the FDA (Food and Drug Administration), and contributing companies from the health industry. ADNI procedures follow a detailed protocol. Cognitive performance of the participants was assessed with the Alzheimer’s Disease Assessment Scale – cognitive subscale (ADAScog; 11 items and modified version with 13 items) [17], the MMSE [18], a number of neuropsychological tests, and the Functional Assessment Questionnaire [19]. ADNI started in 2003 and by the time of our last data download on January 6, 2012 [20], the dataset contained 395 patients diagnosed with MCI at study entry. Two subjects were excluded from our analyses because they had less than 7 years of education.

The NACC (National Alzheimer’s Coordinating Center) project was initiated by the National Institute on Aging /NIH. It developed a large database of standardized clinical and neuropathological research data collected from 29 Alzheimer’s Disease Centers in the USA. Eight of the nine neuropsychological tests used in ADNI are also part of the NACC database [21]. We received data from the freeze of March 19, 2014. We eliminated the data from those participants that were also included in the ADNI project to avoid patients from being considered twice in our analysis. We selected MCI patients with memory impairment, with or without impairment in other domains, who were between 55 and 90 years old and had at least 7 years of education. This left 4328 MCI subjects for our analysis.

The InDDEx (Investigation of Delay of Diagnosis of AD with Exelon®) study [13] was a clinical trial sponsored by the Novartis Pharma, assessing the effect of the cholinesterase inhibitor rivastigmine on disease progression in patients with MCI. This placebo-controlled study did not show evidence of an effect of rivastigmine on either the rate of progression to dementia or the standardized *Z* score for a cognitive test battery. We therefore considered all patients as untreated and included them in our analysis. This conforms with the other four patient samples where dementia-related medication was also permitted. The study applied the ADAScog, a neuropsychological battery that had only verbal fluency (animals) and the Boston Naming Test [22] in common with the battery used in ADNI and a different functional assessment scale. The InDDEx study enrolled 1018 patients randomly assigned to rivastigmine (*n*=508) and to placebo (*n*=510). After exclusions due to missing screening data, missing cognitive data, or age or education outside the admissible range, 861 subjects could be included in the present analysis.

The CNG (Competence Network Germany) study [16, 23] is a longitudinal multicenter cohort study of 14 memory clinics in Germany. It applies the ADAScog (12 subtests), six tests of the Consortium to Establish a Registry for Alzheimer’s Disease – Neuropsychological Assessment Battery (CERAD-NAB) [24] and other tests. We received data of 787 patients with a diagnosis of MCI. After exclusion due to age and education restrictions, 726 were left for our analysis.

The BS-MC (Basel Memory Clinic) sample comprises data of patients referred by practicing physicians to the memory clinic of the University Hospital Basel, Switzerland, for diagnosis and treatment recommendations. Neuropsychological tests include the CERAD-NAB plus Phonemic Fluency (S-words) and Trail Making Tests A and B [25] and Digit Span Forward and Backward. Data of 2135 patients with MCI at baseline were downloaded on September 9, 2016. After application of age and education inclusion criteria data from 1558 patients were left for the analysis.

### Statistical analysis

Demographics and baseline scores of frequently used cognitive tests are summarized in tables and partly in boxplots. To investigate the homogeneity of progression across the five patient samples, we performed meta-analyses for the changes from baseline of cognitive test scores. Confidence intervals in forest plots will show whether there are distinct differences between studies. Measures of heterogeneity confirm these results. Rates of transition from MCI to dementia will be shown in Kaplan-Meier curves, broken down by study, and hazard ratios for age, sex, and education will be compared in proportional hazards models.

## Results

### Definition of MCI

The different inclusion criteria for MCI applied in the five studies are summarized in Table 1. ADNI and CNG used the MMSE to assess cognitive status, although with different inclusion criteria: The lower limit for the MCI was 24/30 in ADNI, but 20/30 in CNG. These two studies requested a Clinical Dementia Rating (CDR) [27] score of 0.5. Cognitive complaints or symptoms (without further specification) were requested in ADNI, NACC, and CNG. ADNI used thresholds in Logical Memory II-dependent on years of education. InDDEx requested a delayed recall score in the NYU-delayed paragraph recall [28] of less than 9, CNG and BS-MC requested at least one cognitive domain below −1 SD (CNG) or −1.28 SD (BS-MC). Patients with major depression were excluded in ADNI, InDDEx, and BS-MC. It will be noted that there are wide differences between the five samples with regard to almost all inclusion and exclusion criteria. It should also be mentioned that almost all datasets contain patients who did not fulfill one or more of their own study’s inclusion and/or exclusion criteria (see comments to Table 3).

**Table 1:** Inclusion and exclusion criteria for the diagnosis of MCI used in five studies

	Eligibility			Inclusion		Exclusion	
	Age	MMSE	Other, medication	Functional impairment	Cognitive impairment	Depression	Other/vascular
ADNI	55–90	24–30	Stable medication, AChEIs, memantine admitted, 6 grades education or work history	No functional impairment, but many with high FAQ scores. CDR=0.5; memory ≥ 0.5	Memory complaint LogMem II, dependent on education	Geriatric Depression Scale ≥6	Hachinski Ischemic Score IS >5
NACC	--	--	Similar to ADNI	Essentially normal daily functions	Cognitive complaint, cognitive decline (clinician's diagnosis)	Not specified	Not specified
InDDEx	55–85	--	No AChEI in previous 2 weeks, no rivastigmine in previous 4 weeks	Cognitive symptoms (not specified); CDR=0.5	NYU delayed paragraph recall<9	HDRS>12, HDRS item1 > 1, DSM-IV major depression	AD criteria from DSM-IV or NINCDS-ADRDA mod. Hachinski Ischemic Score>4
CNG	≥ 50	≥ 20	A broader definition of MCI was used	Complaint of cognitive deficit in daily living; minor changes were tolerated: B-ADL< 4	Decline of cog. abilities (>1 SD) in at least one neuropsychological domain	Not specified	Not specified
BS-MC	N/A	N/A	Consecutively referred patients from GPs	Essentially Winblad et al. [26] criteria; no significant functional decline	Impairment (≤ −1.28 SD; age-, education-,and gender-adjusted) in ≥ one cognitive domain	Probable cause for MCI other than early AD, based on comprehensive medical exam and neuroimaging results	Not specified

### Cognitive tests

A selection of tests, most of them applied in at least two studies, is listed in Table 2. Each study applied a different set of tests. The Mini Mental Status Examination (MMSE) and the Verbal Fluency Test (animals) were the only instruments used in all studies. The ADAScog [17] was applied, although with different modifications, in ADNI, InDDEx, and CNG. Eight of nine tests of a neuropsychological battery used in ADNI were applied in NACC as well, but the Auditory Verbal Learning Test was omitted. Moreover, several procedural details of the Logical Memory delayed recall from the Wechsler Memory Scale (WMS) differed significantly in ADNI and NACC (details in [20]). The Boston Naming Test [22] was performed in ADNI and NACC with 30 items, but with only 15 items in CNG and BS-MC. The Digit span forward and backward and the Trail Making Test A and B [29] were applied in all studies except in InDDEx; however, the Trail Making Tests were conducted with different time limits (e.g., for TMTA: 150 s in NACC and CNG, 180 s in BS-MC). The Clock Drawing Test was applied in all studies except in NACC, but different scoring methods were used. The Clinical Dementia Rating scale was applied in all studies but BS-MC. Three different versions of functional assessment were used. The CERAD battery was only used in CNG and BS-MC. A few other tests were applied in only one study.

**Table 2:** List of selected cognitive tests applied in five studies

Test	ADNI	NACC	InDDEx	CNG	BS-MC
ADAScog 11 and modified	11 & mod.		11 & mod	11	
Logical Memory II	x	x		x	
Digit Span Forward	x	x		x	x
Digit Span Backward	x	x		x	x
Category Fluency, Animals	x	x	x	x	x
Category Fluency, Vegetables	x	x			
Trail Making Test B	x	x		x	x
Boston Naming Test	x	x	x^a^	x	x
Auditory Verbal Learning Test	x				
Digit Symbol	x	x			
Trail Making Test A	x	x		x	x
Clock Drawing Test	x		x	x	x
Functional Assessment	x	x	ADCS-ADL	Bayer-ADL	
Logical Memory I	x			x	
American National Adult Reading Test	x				
Clinical Dementia Rating	x	x	x	x	
Mini Mental Status Examination	x	x	x	x	x
Phonemic fluency, S-words					x
CERAD Wordlist + intrusion errors / savings				x	x
CERAD constructional praxis				x	x
Neuropsychiatric Inventory (NPI)	x	x	x	x	
Digit cancellation task			x		

^a^Only 85 values

### Demographics (Table 3)

Patients in ADNI and NACC were oldest (74.2±7.5 and 74.4±7.9), those in InDDEx and BS-MC were younger (70.1±7.6 and 69.7±9.1), and CNG (68.0±7.9) comprised the youngest sample. Duration of education was highest in ADNI (15.7±3.0 years) and NACC (15.2±3.0 years) and lowest in InDDEx (11.8±3.5). CNG (12.3±2.8) and BS-MC (12.0±3.1) were in between. The proportion of females was lowest in ADNI (35.7%), highest in NACC (48.8%) and InDDEx (49.0%), and intermediate in CNG (45.7%) and BS-MC (45.8%). The proportion of patients with two ApoE4 alleles was highest in ADNI (11.9%), intermediate in NACC (8.7%), InDDEx (9.6%) and BS-MC (9.4%), and lowest in CNG (5.4%).

**Table 3:** Descriptive statistics of demographics and baseline scores MMSE, Verbal Fluency (animals), and ADAScog (11 subtests)

	ADNI *n* = 395	NACC *n* = 4328	InDDEx *n* = 831	CNG *n* = 726	BS-MC *n* = 1558
Age ($\overline{x}\pm SD$)	74.2±7.5	74.4±7.9	70.1±7.6	68.0±7.9	69.7±9.1
Education ($\overline{x}\pm SD$)	15.7±3.0	15.2±3.0	11.8±3.5	12.3±2.8	12.0±3.1
Female, *n* (%)	141 (35.7)	2113 (48.8)	422 (49.05)	332 (45.7)	713 (45.8)
ApoE4^a^ 1 allele no (%) 2 alleles no (%)	n_ApoE_=395 165 (41.8) 47 (11.9)	n_ApoE_=2523 951 (37.7) 219 (8.7)	n_ApoE_=396 131 (32.3) 39 (9.6)	n_ApoE_=577 200 (34.7) 200 (34.7)	n_ApoE_=53 22 (41.5) 5 (9.4)
MMSE ($\overline{x}\pm SD$) Min^b^-max	27.0±1.8 23–30	27.0±2.4 2–30	27.2±2.5 16–30	27.1±2.1 17–30	27.4±2.3 14–30
Verb. Fl. ($\overline{x}\pm SD$) Min-max	15.9±4.9 5–30	16.0±5.0 0–35	17.5±5.9 2–38	17.5±5.5 3–32	17.4±5.5 3–38
ADAScog ($\overline{x}\pm SD$) min-max	11.5±4.4 2–27.7	-	10.0±4.7 1–27	11.7±5.1 0–35	-

^a^ApoE was not determined in all patients, number of evaluations is given as *n*_ApoE_, percent of ApoE evaluations shown in parentheses. ^b^MMSE is below inclusion criterions 24 in ADNI (*n*=1) and below 20 in CNG (*n*=5)

### Baseline data

**Fig. 1:**
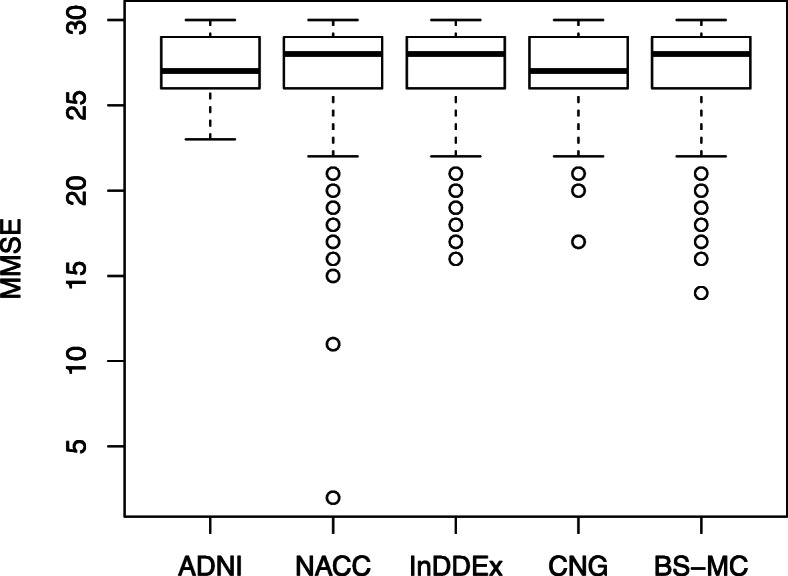
Boxplot of Mini Mental Status Examination (MMSE) scores at baseline in each study

**Fig. 2:**
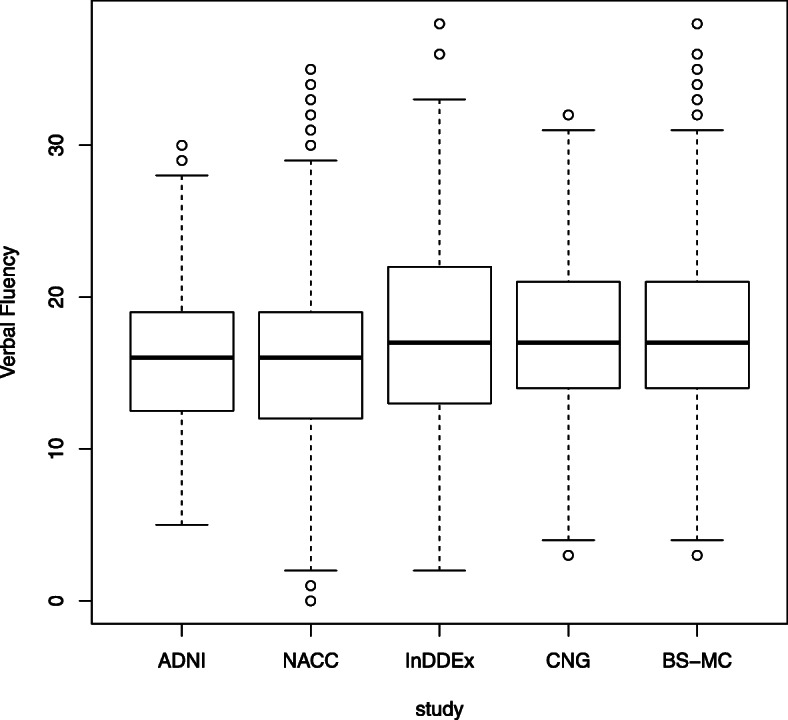
Boxplot of Verbal Fluency scores (animals) at baseline in each study

In each study, the quartile range of MMSE is from 26 to 29 (Fig. 1), with several outliers in NACC, InDDEx, CNG, and BS-MC, displaying much lower values that would preclude a diagnosis of MCI. Only ADNI had no outliers, because the minimum score of 24 was an inclusion criterion. Verbal fluency is 1.4 to 1.6 points higher in InDDEx, CNG, and BS-MC compared to ADNI and NACC (Table 3, Fig. 2). InDDEx patients performed better on the ADAScog (10.0±4.7) than CNG patients (11.7±5.1), but ADNI patients (11.5±4.4) performed similar to CNG patients. In InDDEx, ADNI, and CNG, there were patients with ADAScog values typical of dementia rather than MCI. In the Boston Naming Test [22], patients of ADNI and NACC achieved on average about 25 of 30 items, while patients of CNG and BS-MC achieved about 13.4 of 15 items. Results on the Trail Making test cannot be compared because of different time limits used.

### Progression of cognitive scores

**Fig. 3:**
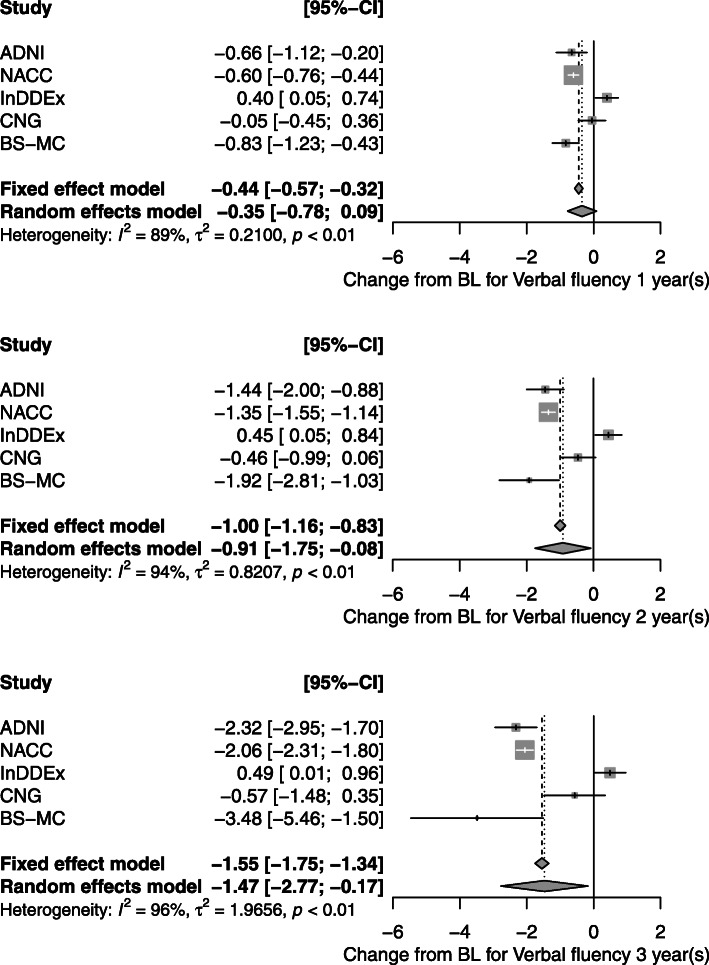
Forest plots for the change of Verbal Fluency (animals) from baseline to 1, 2, and 3 years. Mean changes and 95% confidence intervals for each study and for the overall effect in the fixed effects and the random effects model are given. *τ*^2^ is the between-study variance, *I*^2^ measures heterogeneity (between study variance over total variance), *p* value for the test of heterogeneity. Graphs show study specific means and confidence intervals for each study as gray squares and lines and for overall effects as diamonds. Size of squares represents precision of individual treatment estimates

Verbal fluency (animals) is—next to the MMSE—the only test score available in all five studies. From baseline to 1 year, InDDEx patients improved on average by 0.4 words, CNG patients remained stable, but ADNI, NACC, and BS-MC patients worsened by 0.6, 0.7, and 0.8 words, respectively (Fig. 3). Confidence intervals for InDDEx and the latter three studies are disjoint. The differences between studies increased for the 2- and 3-year follow-up: InDDEx subjects still improved by 0.5 words, the latter three worsened by up to 2.1 (NACC) and 3.5 (BS-MC) words after 3 years (Fig. 3).

ADAScog worsened considerably in ADNI, slightly in CNG, but improved slightly in InDDEx. Boston Naming Test scores show similar decline in ADNI, NACC, and BS-MC (in relation to the number of items), while the decline in CNG is very small. The CERAD-word list (delayed recall) worsened considerably in BS-MC, but remained unchanged in CNG.

### Transition from MCI to dementia

**Fig. 4:**
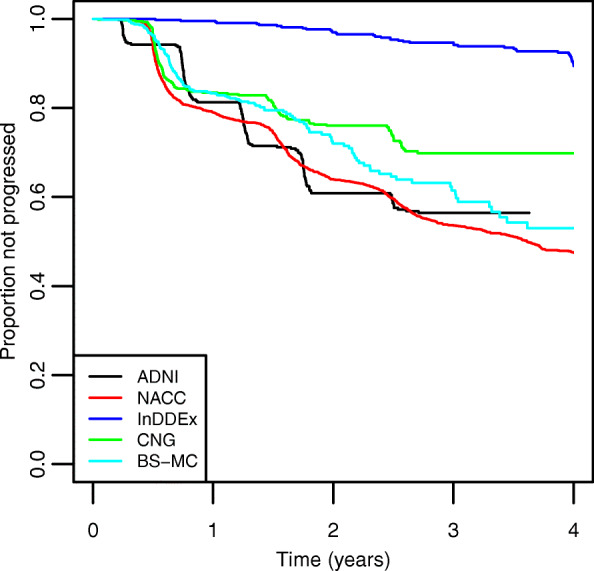
Kaplan-Meier plots of the proportion progressed from MCI to dementia versus time for each study

Time to transition from MCI to dementia was usually ascertained at scheduled visits. Sometimes, however, patients were examined in an extra visit and then classified as being demented. Kaplan-Meier curves for the time to transition from MCI to dementia (mostly AD) are shown in Fig. 4. They confirm that the InDDEx patients were in a particularly stable state. Continuation with an MCI diagnosis was considerably less frequent in the CNG sample, while ADNI and NACC patients were the fastest to progress. Transition rates after 3 years are between 5.9% (InDDEx) and 46.4% (NACC), both with a standard error of 1.1%.

**Fig. 5:**
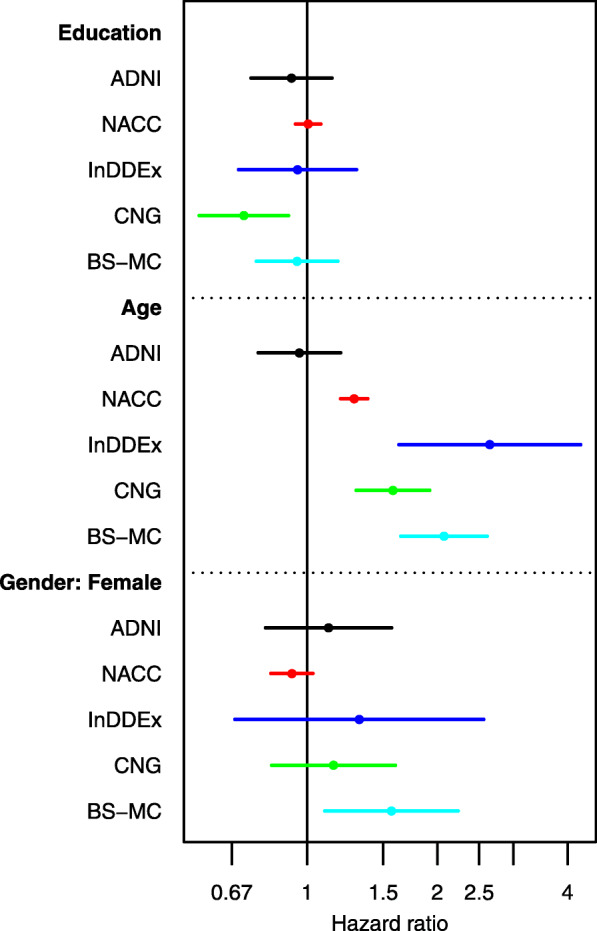
Hazard ratios of five Cox proportional hazard regression models for each study. For education the hazard ratio for progression to dementia is shown for an increase of 4 years, for age, it is shown for an increase of 10 years, for gender, it is for females relative to males

Cox regression models with covariates years of education, age, and sex were estimated for each study. Hazard ratios and 95% confidence intervals are shown in Fig. 5. The hazard ratio for education was 0.71 (for 4 years) in CNG and close to 1 in the other studies. The hazard ratio for age was close to 1 in ADNI and distinctly positive in the other studies. The hazard ratio for females was 1.56 in BS-MC and close to 1 in the other studies.

## Discussion and conclusion

The PGSA (Placebo Group Simulation Approach) was submitted “for debate” to the Alzheimer community [1]. The novel study design was intended primarily to resolve an ethical problem of prevention trials involving aged subjects at risk of shifting from a pre-symptomatic into a dementia stage: the long-term use of placebo typical of RCTs (randomized controlled trials). Accordingly, instead of using a concomitant placebo group for comparison with a hopefully effective novel treatment, the PGSA applies mathematical algorithms to forecast the expected outcomes of pre-symptomatic AD patients from their baseline data and to compare those with the outcomes on experimental treatments. The PGSA was deemed to “have an advantage over the use of historical controls in futility designs in that it is based on the patient’s own observed clinical features.” [30].

Our published algorithms were derived from the ADNI database (http://www.loni.ucla.edu/ADNI) [31] that contained anamnestic, biological, neuroimaging, and neuropsychological findings from 397 North American patients with a diagnosis of MCI. Our analyses highlighted the strong impact of neuropsychological performance data recorded at baseline, in addition to information from subjects’ history such as age, sex, and education, on MCI disease trajectories in the following years. A first attempt at validation of the PGSA algorithms using data from an independent MCI database (NACC; https://www.alz.washington.edu) confirmed the importance of neuropsychological performance data recorded at baseline to forecast cognitive decline in MCI [20]. However, we also noted that there was some slight over-estimation of cognitive decline when the ADNI-based PGSA algorithms were applied to the NACC MCI dataset for a follow-up of more than 2 years. This observation led to the question as to whether the published PGSA algorithms could be confidently applied to other longitudinal MCI data.

The current analysis comprised three MCI databases in addition to the ones from ADNI and NACC. One of these three originated from an RCT with a cholinesterase inhibitor sponsored by a drug company [13] and two from clinical case series: one from a network of memory clinics in Germany [23], the other one from a single memory clinic in Basel, Switzerland [32]. The five databases contained information on 395 (ADNI) up to 4328 (NACC) individuals. Although all patients had a diagnosis of MCI, inspection of Table 1 shows that inclusion and exclusion as well as other eligibility criteria varied considerably between the five samples. Wide differences also existed between the five databases with regard to the neuropsychological scales and instruments used at baseline and at follow-up to document the changes in patients’ cognitive performance (Table 2). Only the MMSE and the Verbal Fluency Test with “animals” were applied throughout.

Despite the differences in inclusion and exclusion criteria, the demographic and even more so the baseline cognitive performance data of the five patient samples were not as variable as one might expect. While patients’ mean ages varied between 68.0 (CNG) and 74.4 (NACC) years and the ADNI sample contained a lower percentage of female participants than the other four, mean MMSE scores at baseline varied only minimally (Table 3, Fig. 1), and the same was seen with regard to the ADAS scores in the three studies where this scale was used. Nevertheless, judging from their MMSE and/or ADAScog scores, a number of patients in the NACC, InDDEx, CNG, and BS-MC samples should be classified as being demented rather than having MCI. Interestingly, the participants of InDDEx, CNG, and BS-MC had slightly better performance on the Verbal Fluency Test than those of ADNI and NACC (Table 3, Fig. 2), although the latter had benefited from more years of education on the average.

In contrast to the similarity of their cognitive performance data noted at baseline, the five samples differed markedly with regard to their subsequent cognitive performance and clinical development. As seen in Fig. 3, scores on the Verbal Fluency Test behaved differently in the InDDEx than in the other four groups: with increasing study duration performance deteriorated continuously in the ADNI, NACC, CNG, and BS-MC samples, but were slightly improved from baseline after 1 year and then stayed stable in the InDDEx group. An even greater disparity is seen with regard to the number of transitions from MCI to dementia (Fig. 4): Whereas some 90% of the InDDEx patients did not progress to dementia within the 4 years of observation, the respective percentages were around 70 for CNG, around 60 for ADNI and BS-MC and somewhat above 50 for NACC. What could be an explanation of these big differences?

First, one should note that the difference in transition rates between the ADNI and the NACC participants is small. This is not a surprise given that both MCI patient samples were collected in North America and that the ADNI sample partly constituted a selection from the large NACC data collection. Thus, it is likely that both the MCI inclusion/exclusion and the transition criteria applied to the ADNI and the NACC data were similar. Transition rates for the BS-MC sample were also close to the ones seen in ADNI and NACC, suggesting that the inclusion/exclusion criteria for MCI and the criteria to diagnose transition to dementia were applied similarly in the Basel and in the North American centers. More difficult to understand are the lower transition rates in CNG and, particularly, in InDDEx. The latter dataset differed from the other four in that InDDEx was not a case series from one or more memory clinics but a clinical drug trial sponsored by a pharmaceutical company and carried out in 12 different countries in Europe, South Africa, South America, and in the USA. While one cannot exclude that this geographic variety (or perhaps some investigators’ desire to include as many patients as possible) compromised proper selection of MCI patients, it is of note that other, although shorter, company-sponsored drug studies carried out in the same decade as InDDEx also differed markedly with regard to transition rates from MCI to dementia. Thus, Petersen et al. [8] reported annual transition rates of 16% in a 3-year study with donepezil and vitamin E, and relatively high percentages of transitions were also seen in two separate 2-year studies with galantamine [15]. In contrast, an RCT with a selective COX-2 inhibitor noted rather low annual transition rates: 6.4% on rofecoxib, 4.5% on placebo [33]—although these rates were still higher than the one reported in [13].

Whatever the reasons for the varying transition rates in these mostly company-sponsored trials are, it is obvious that the MCI criteria available some 20 years ago were not sufficiently precise to allow selection of clinically homogeneous and internationally comparable MCI patient groups. Ward et al. [34] and Han et al. [35] noted that—owing to the fuzzy boundaries between normal, MCI and dementia—all available estimations of the incidence and prevalence of MCI varied widely. Edmonds et al. [36] and Stephan et al. [37] stressed that samples of non-specified MCI cases will include individuals with different brain pathologies, leading to widely different clinical trajectories. As a consequence thereof, the predictive power of MCI diagnoses is poor, allowing, e.g., up to 59% of patients with MCI to revert to normal within up to 17 years after a first diagnosis [38]. The problem to delineate MCI properly was noted early on [9, 39], and efforts were made to ameliorate the situation (e.g., [26]). Nevertheless, ambiguity remained: Although an international expert group [10] stressed the importance of determining whether there is objective evidence of cognitive decline and recommended cognitive testing for quantitatively assessing the degree of cognitive impairment for a diagnosis of MCI, these authors also emphasized that normative ranges of neuropsychological tests (typically those listed in Table 2) “are guidelines and not cutoff scores.” ([10] p. 272). In contrast to this position, it is our opinion that the scientific community needs to agree on age- and education-adjusted cutoff scores in order to make MCI a scientifically useful concept and to ensure that study results from different sources will be comparable. It simply cannot be that individuals with MMSE scores of less than 20 or ADAScog scores of more than 20 be included in so-called MCI patient samples (Table 3).

Another critical issue is the use of specific neuropsychological tests: as noted in the current analyses, only two tests were part of all five studies considered, making comparison between patient samples virtually impossible. Moreover, for several tests, different versions and scoring systems exist and are being used. This is another impediment when one tries to compare results between studies. In the USA, a series of Uniform Data Sets (UDS) have been proposed in [40] to improve this unsatisfactory situation. A separate issue refers to the underlying disorder of MCI and subsequent dementia. While neuropsychological test performance with an internationally agreed set of tests and cutoffs will allow for the determination of cognitive deficits, specific biomarker requirements would also allow to describe pathophysiologically more homogenous groups of patients with MCI, e.g., MCI due to AD. As of today, and as shown in our analyses, the heterogeneity at all these levels is way too big to allow meaningful conclusions with regard to the scientific merits of algorithm-based approaches such as the PGSA.

The scientific rationale of our earlier studies [1, 15] was to make use of well-defined historical data in clinical treatment or prevention trials. Inclusion of historical information for comparison with current treatment data has been discussed since more than 40 years ([41] and later references in [7]). Suggestions range from performing a single arm study which is compared to the historical control, over integrating historical control data with new controls—up to avoiding any use of historical data completely. Different options are available to integrate historical data [3]: (1) pooling them with new controls, (2) testing for differences between historical and new controls and pool them only if no differences are detected, (3) down-weighting the historical data by power priors dependent on the discrepancy between observed and historical control data, (4) choose a prior distribution for the means of the historical and the new control groups and apply a Bayesian model (see [3] for details on these methods), and (5) perform a random effects meta-analysis of the historical controls and down-weight their sample size according to the between-study variation [7].

In any case, data pooling is only permissible if the historical controls are exactly equivalent to the new control. This may hold for a set of pharmaceutical studies with basically the same protocol and equivalent patient populations. In less narrowly defined circumstances, this is rarely the case, due to different populations and more or less different inclusion and exclusion criteria. Down-weighting of historical data takes heterogeneity into account. It decreases the weight of the historical data from the total number of patients to a smaller number which is called the *prior effective sample size* [7]. For the five studies considered in the current analysis, the 2144 patients for whom data for change of verbal fluency after 3 years were available, would be down-weighted to 10 patients, according to formulas [7]. However, all methods of integrating historical controls are only valid under the assumption of exchangeability, i.e., if no systematic differences exist between the control groups [7, 42]. In view of the distinctly apart confidence intervals in Fig. 3, exchangeability cannot be assumed for the patient samples considered here. This makes all attempts to integrate these data in new studies futile. Insofar, our current failure of forecasting algorithms is consistent with theoretical considerations on using prior information.

### Limitations

Drop-outs are a common problem in long-term clinical studies. In the five studies considered, the rate of drop-outs after 3 years was quite different: 70% in NACC, 37% in ADNI, 25% in InDDEx, 77% in CNG, and 98% in BS-MC. While studies with a stricter visit regimen (in our case InDDEx and ADNI) have lower drop-out, convenience samples such as CNG and BS-MC tend to have very high drop-out rates. This factor may cause bias and could make studies less comparable. For example, one might assume that convenience samples contain a larger number of frail patients than samples in controlled studies, that frail patients drop-out with higher probability, and that, as a consequence, the results of cognitive tests would worsen less in convenience samples. However, our results do not support this hypothesis: Patients of the InDDEx study (with the lowest dropout rate) improved their cognitive results after 2 and 3 years, whereas patients in BS-MC showed the most pronounced decrease (cf. Fig. 3). CNG and NACC, the two studies that collected data from Alzheimer’s coordinating centers and/or memory clinics, also showed distinctly different changes (Fig. 3). Transition rates in the InDDEx sample were very low, and the transition rate of the CNG sample was between InDDEx and the other studies (cf. Fig. 4). This conforms to the cognitive test results, but, again, does not support the hypothesis concerning bias due to drop-out.

Transitions to dementia were in most studies ascertained at more or less strictly scheduled visits. We nevertheless applied Kaplan-Maier and Cox Regression analysis assuming continuous time. Figure 4 shows the pattern of event times for strict visit schedules of ADNI and InDDEx and less clearly for NACC and CNG. The true curves would interpolate between the top right bends of the curves in Fig. 5, but this would not change the interpretation of the transition rates.

### Final remark

A basic requirement of the PGSA and similar approaches is the availability of uniformly collected, high-quality data of the respective population, in our case patients with MCI. Our analysis of five differently assembled MCI datasets shows that this requirement is not met, and access to other, more recently collected databases have proven to be somewhat cumbersome. As pointed out by one of the reviewers of this paper, the availability of data in the AD field is low and this “is really problematic. In such a dreadful disease, data should be made available to any researcher in an effort to produce harmonized and robust modeling of the disease….We desperately need an open science approach in our field in which both academia and the industry would participate by sharing anonymized data. The ADNI model and thousands of publications it allowed is an indication of how much that is needed.” We definitely agree with this statement.

## Data Availability

The data that support the findings of this study are available from Alzheimer‘s Disease Neuroimaging Initiative (ADNI; http://www.loni.ucla.edu/ADNI), National Alzheimer’s Coordinating Centers (NACC; https://www.alz.washington.edu), Novartis AG, Basel, Competence Network Germany [23], and University Departement of Geriatric Medicine FELIX-PLATTER (A.U.M), but restrictions apply to the availability of these data, which were used under license for the current study, and so are not publicly available. Data are however available from the authors upon reasonable request and with permission of third parties mentioned before.
